# Overcoming amplification-mediated resistance to sotorasib by dose re-escalation in *KRAS G12C* mutant NSCLC: a case report

**DOI:** 10.1093/oncolo/oyaf030

**Published:** 2025-03-20

**Authors:** Antonio Vitale, Emanuele Vita, Alessio Stefani, Alessandra Cancellieri, Filippo Lococo, Giampaolo Tortora, Emilio Bria

**Affiliations:** Comprehensive Cancer Center, Medical Oncology Department, Fondazione Policlinico Universitario Agostino Gemelli IRCCS, 00168 Rome, Italy; Faculty of Medicine and Surgery, Università Cattolica del Sacro Cuore, 00168 Rome, Italy; Comprehensive Cancer Center, Medical Oncology Department, Fondazione Policlinico Universitario Agostino Gemelli IRCCS, 00168 Rome, Italy; Faculty of Medicine and Surgery, Università Cattolica del Sacro Cuore, 00168 Rome, Italy; Comprehensive Cancer Center, Medical Oncology Department, Fondazione Policlinico Universitario Agostino Gemelli IRCCS, 00168 Rome, Italy; Faculty of Medicine and Surgery, Università Cattolica del Sacro Cuore, 00168 Rome, Italy; Pathology Unit, Fondazione Policlinico Universitario Agostino Gemelli IRCCS, 00168 Rome, Italy; Faculty of Medicine and Surgery, Università Cattolica del Sacro Cuore, 00168 Rome, Italy; Thoracic Surgery Unit, Fondazione Policlinico Universitario Agostino Gemelli IRCCS, 00168 Rome, Italy; Comprehensive Cancer Center, Medical Oncology Department, Fondazione Policlinico Universitario Agostino Gemelli IRCCS, 00168 Rome, Italy; Faculty of Medicine and Surgery, Università Cattolica del Sacro Cuore, 00168 Rome, Italy; Comprehensive Cancer Center, Medical Oncology Department, Fondazione Policlinico Universitario Agostino Gemelli IRCCS, 00168 Rome, Italy; Faculty of Medicine and Surgery, Università Cattolica del Sacro Cuore, 00168 Rome, Italy; Medical Oncology, Ospedale Isola Tiberina – Gemelli Isola, 00186 Rome, Italy

**Keywords:** *KRAS G12C*, sotorasib, acquired resistance, case report

## Abstract

Precision oncology has transformed non-small cell lung cancer (NSCLC) treatment by tailoring therapies to the genomic profile of the disease, significantly improving clinical outcomes. However, acquired resistance to molecularly targeted therapies remains a major challenge. This report details a 69-year-old woman with *KRAS G12C*-mutant metastatic NSCLC who developed resistance to sotorasib, a *KRAS G12C* inhibitor. Initially responding to the standard dose of 960 mg, the patient required a dose reduction to 480 mg due to liver toxicity. After 20 months, oligoprogression occurred, managed through surgical resection. Molecular analysis of the resected tissue identified *KRAS* amplification as a resistance mechanism. Following disease progression, re-escalation of sotorasib to 960 mg led to renewed tumor response without additional toxicity. This case highlights dose re-escalation as a potential strategy to address resistance in selected patients and underscores the critical role of molecular profiling and personalized approaches in optimizing targeted NSCLC treatments.

**Key points**

- *KRAS G12C* mutation accounts for 10%-13% of advanced non-squamous NSCLC cases. *KRAS G12C*-specific inhibitors demonstrated significant clinical efficacy in NSCLC patients harboring this mutation and have recently been approved after at least one prior line of systemic therapy.
- Liver toxicity is a frequent adverse event in patients receiving targeted therapies after immunotherapy. This issue often necessitates dose reductions, which may compromise drug efficacy.
- New molecular profiling at the time of disease progression is a valuable tool to identify resistance mechanisms and design personalized treatment strategies to potentially overcome these barriers and restore clinical benefit.
- To the best of the authors’ knowledge, this is the first documented case demonstrating the safety and efficacy of re-escalating the dose of sotorasib after a prior dose reduction due to liver toxicity. This approach effectively managed resistance caused by target amplification, achieving long-term disease control without additional toxicity.

## Introduction

Kirsten rat sarcoma (*KRAS*) is the most commonly mutated oncogene in advanced, non-squamous, non-small cell lung cancer (NSCLC), with the *G12C* mutation being the most prevalent variant (40%), accounting for 10%-13% of advanced non-squamous NSCLC cases.^1^ Recent breakthroughs transformed the previously undruggable *KRAS* into a viable therapeutic target. Two specific *KRAS G12C* inhibitors, adagrasib and sotorasib, showed significant clinical activity in NSCLC patients harboring such mutation, achieving response rates of 43% and 37% in later treatment lines, respectively.^2,3^ Despite these promising results, the durability of response remains a concern, as most patients eventually develop resistance and experience disease progression.^4^ Several mechanisms of resistance to *KRAS G12C* inhibitors have been identified and broadly categorized into on-target and off-target. On-target resistance often involves acquired *KRAS* alterations, or high-level amplification of the *KRAS G12C* allele. Off-target mechanisms encompass *MET* amplification, oncogenic fusions involving *ALK*, *RET, BRAF, RAF1*, and *FGFR3*, as well as loss-of-function mutations in *NF1* and *PTEN*.^5^ Although these molecular pathways have been extensively described in the literature, effective strategies to overcome resistance remain elusive. In this context, we present a unique case report of a patient with *KRAS G12C*-mutant metastatic NSCLC who developed resistance to sotorasib after a dose reduction due to liver toxicity—a common issue in patients previously treated with immunotherapy before *KRAS G12C* inhibitors. This case highlights dose re-escalation as a potential strategy to counteract resistance mediated by target amplification, restoring disease control and improving clinical outcomes.

## Case Presentation

In April 2020, a 69-year-old woman presented with night fever and persistent cough. She was a non-smoker without any known exposure to pneumotoxic agents. Her medical history was significant for early-stage breast cancer, which had been treated with surgery, radiation therapy, and 5 years of adjuvant tamoxifen. Computed tomography (CT) and positron emission tomography (PET-CT) revealed a mass in the lower lobe of the right lung. The patient underwent right lobectomy with mediastinal lymphadenectomy and was diagnosed with stage IIIA lung mucinous adenocarcinoma (pT4, pN0, cM0). Polymerase Chain Reaction (PCR—EasyPGX ready *KRAS*, Diatech Pharmacogenetics, Jesi, Italy) revealed a *KRAS* c.34G > T p.G12C mutation while immunohistochemistry (IHC—antibody 22C3 pharmDx, Dako, Santa Clara) for Programmed Death Ligand 1 (PD-L1) tested positive in 5% of tumor cells. No further genomic aberrations were detected in *NRAS*, *BRAF*, *EGFR*, *ROS1,* or *ALK* genes. After thoracic surgery, adjuvant chemotherapy with cisplatin and vinorelbine was early discontinued due to grade 3 neuropathy. Follow-up showed no recurrence for 1 year until May 2021, when a PET-CT identified disease relapse with multiple hypermetabolic lung nodules. Based on the molecular profile, the patient started first-line therapy with carboplatin AUC5, pemetrexed 500 mg/m^2^, and pembrolizumab 200 mg, achieving disease control for 8 months. Upon disease progression in January 2022, second-line sotorasib at the standard dose of 960 mg daily was initiated. After 2 months, the patient experienced grade 2 elevation of alanine transaminase (ALT) and aspartate transaminase (AST), which did not resolve after a 2-week drug interruption. Consequently, after hepatoxicity resolution to grade 1, the dose was reduced to 480 mg daily without any further alterations of liver tests.

### Molecular Tumor Board

**Figure 1.**
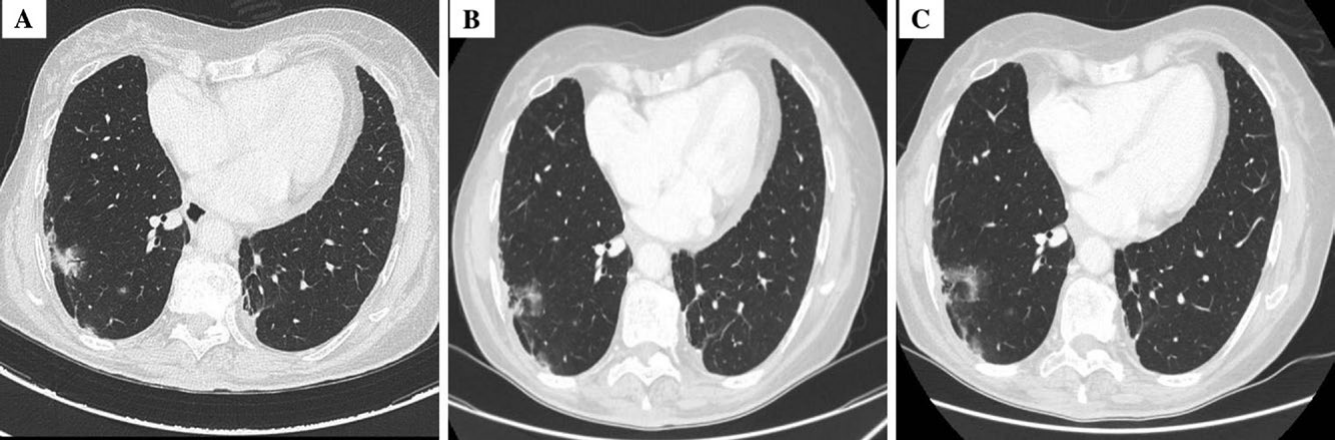
CT-scan sections focusing on the largest measurable lesion in the upper lobe of the right lung. (a) Disease progression on March 2024. (b) Restored tumor response after Sotorasib dose re-escalation in July 2024. (c) Confirmed tumor response on October 2024. Abbreviation: CT, computed tomography.

By September 2023, after 20 months of treatment, a single lung nodule showed progression while overall disease remained stable. Then, a wedge resection was performed on the progressing lesion to manage oligoprogression and the surgical specimen underwent Next-Generation Sequencing with Oncomine Precision Assay (Thermo Fisher Scientific) revealing acquired *KRAS* amplification (6.7 copies) alongside the original *p.G12C* mutation (*c.34G > T*, VAF 69.5%). Sotorasib at 480 mg was resumed post-surgery until March 2024, when disease progression occurred in multiple lung nodules (Figure 1a). Given the availability of a molecular characterization at the time of progressive disease, the case was evaluated by our institutional molecular tumor board to determine whether a tailored targeted strategy could be pursued. After a careful evaluation of the balance between the risk of toxicity and the awaited clinical benefit, the board decided to re-escalate the dose of sotorasib to 960 mg daily, hypothesizing that *KRAS* amplification was driving resistance and that the reduced dose might be insufficient to achieve an effective target inhibition.

### Patient Update

**Figure 2.**
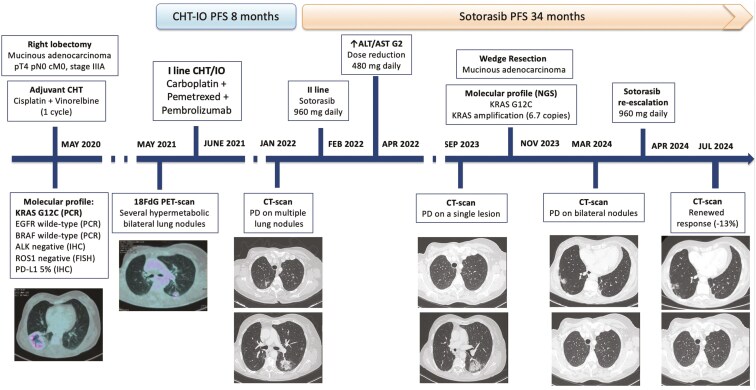
Timeline of the clinical history reporting treatment sequence, local therapies, and molecular assessments. Abbreviations: 18FdG, PET, 18-Fluorodeoxyglucose, Positron, Emission, Tomography, ALT, Alanine, Transaminase,; AST, Aspartate, transaminase,; CHT, Chemotherapy,; CT, Computed, Tomography,; FISH, Fluorescence, In, Situ, Hybridization,; IHC, Immunohistochemistry,; IO, Immunotherapy,; NGS, Next, Generation, Sequencing; PCR, Polymerase, Chain, Reaction.

Three months after re-escalation, the patient experienced a 13% reduction in the total diameter of the lung lesions evaluated according to RECIST criteria v.1.1^6^ (Figure 1b), confirmed by further imaging in October 2024 (Figure 1c). Notably, the higher dose was well-tolerated, with liver function tests within normal ranges. As of this report, the patient continues to benefit from sotorasib with no significant toxicities (Figure 2).

## Discussion

Dose reduction due to liver toxicity is a common challenge in patients receiving targeted therapies, particularly those previously treated with immunotherapy. This is a substantial issue for *KRAS G12C*-mutant patients, as most of them receive PD-(L)1 inhibitors as first-line treatment.^7,8^ A key concern with *KRAS G12C* inhibitors is the increased toxicity associated with higher doses. In the case of sotorasib, a dose-ranging study comparing 960 mg with 240 mg showed that the higher dose led to significantly more grade 3 treatment-related adverse events, resulting in more frequent dose reductions and interruptions. Despite this, no significant improvement in efficacy was observed at the higher dose, and pharmacokinetic data showed that increasing the dose did not lead to higher plasma concentrations, as the drug’s absorption appears to be saturable. Although this is compelling evidence, approvals from regulatory agencies still consider 960 mg as the standard dose of sotorasib, but the debate is still open on which is the best dose to balance efficacy and toxicity.^9^

Target amplification is a well-established resistance mechanism to various targeted therapies, including *KRAS G12C* inhibitors. Previous studies showed that full doses of targeted inhibitors typically result in high selective pressure leading to gatekeeper mutations; in contrast, the target amplification often arises in patients exposed to intermediate drug doses, such as those who experienced dose reduction due to toxicity.^10^

In this case, the rationale for dose re-escalation was based on the premise that *KRAS* amplification could result in suboptimal target inhibition, especially in patients exposed to lower doses. Increasing the dose may enhance anti-*KRAS G12C* activity, potentially overcoming resistance driven by the amplified target protein and restoring anti-tumor efficacy. A similar strategy has been validated in patients with chronic myelogenous leukemia. Clinical trials demonstrated that increasing the dose of imatinib could restore effectiveness in patients who developed resistance due to *BCR-ABL* gene overexpression, resulting in durable hematologic and cytogenetic responses.^11,12^ Our findings align with this evidence, suggesting that increasing drug exposure may counteract *KRAS* amplification and highlighting the importance of molecular profiling at the time of disease progression, both on tissue or liquid biopsy, to identify resistance mechanisms and design tailored treatment approaches.^13,14^

The risk of toxicity from dose re-escalation must be considered. Several experiences are reported about the safety of full dose restoration in patients who developed drug-related toxicities showing tolerable and manageable profiles.^15-18^ Consistent with the literature, our patient tolerated the higher dose well, despite prior liver toxicity. This emphasizes the potential of personalized dosing strategies and the importance of a careful monitoring of adverse events, suggesting that some patients may develop tolerance to drug-related toxicities over time, allowing for safe dose re-escalations when clinically indicated.

While dose escalation shows promises, it is essential to acknowledge its limitations. This approach may not be effective against all mechanisms of resistance, particularly those involving bypass pathways or alternative oncogenic drivers. Additionally, the balance between efficacy and toxicity remains crucial. Several areas need further investigation to optimize the use of dose escalation in targeted therapies, including predictive biomarkers to identify patients more likely to benefit from this strategy, combination approaches that pair high-dose targeted therapies with inhibitors of potential bypass pathways, and novel drug delivery methods that allow for higher local drug concentrations in tumor tissue while minimizing systemic toxicity.^19,20^

To the best of our knowledge, this is the first reported case of successful dose re-escalation overcoming amplification-mediated resistance in *KRAS G12C*-mutant NSCLC following disease progression after dose reduction due to toxicity, offering valuable insights on this emerging patients’ population as *KRAS G12C* inhibitors are expected to become a standard treatment for a large number of NSCLC patients in the next future.

In conclusion, as precision oncology evolves, adaptive and personalized strategies tailored to the genomic profile of the disease may significantly improve outcomes for molecularly enriched NSCLC patients facing resistance to novel target therapies.

## Data Availability

All the data underlying this article are presented in the article and available from the corresponding author upon reasonable request.

## References

[1] Lim TKH , Skoulidis F, Kerr KM, et al KRAS G12C in advanced NSCLC: prevalence, co-mutations, and testing. Lung Cancer. 2023;184:107293. https://doi.org/10.1016/j.lungcan.2023.107293 37683526

[2] Skoulidis F , Li BT, Dy GK, et al Sotorasib for lung cancers with *KRAS* p.G12C mutation. N Engl J Med. 2021;384:2371-2381. https://doi.org/10.1056/NEJMoa2103695 34096690 PMC9116274

[3] Jänne PA , Riely GJ, Gadgeel SM, et al Adagrasib in non–small-cell lung cancer harboring a *KRAS*^*G12C*^ mutation. N Engl J Med. 2022;387:120-131. https://doi.org/10.1056/nejmoa2204619 35658005

[4] Reck M , Carbone DP, Garassino M, Barlesi F. Targeting KRAS in non-small-cell lung cancer: recent progress and new approaches. Ann Oncol: Off J Eur Soc Med Oncol. 2021;32:1101-1110. https://doi.org/10.1016/j.annonc.2021.06.001 34089836

[5] Awad MM , Liu S, Rybkin II, et al Acquired resistance to KRAS ^G12C^ inhibition in cancer. N Engl J Med. 2021;384:2382-2393. https://doi.org/10.1056/nejmoa2105281 34161704 PMC8864540

[6] Eisenhauer EA , Therasse P, Bogaerts J, et al New response evaluation criteria in solid tumours: Revised RECIST guideline (version 1.1). Eur J Cancer. 2009;45:228-247. https://doi.org/10.1016/j.ejca.2008.10.026 19097774

[7] Ernst SM , Hofman MM, van der Horst TE, et al Hepatotoxicity in patients with non-small cell lung cancer treated with sotorasib after prior immunotherapy: a comprehensive clinical and pharmacokinetic analysis. EBioMedicine. 2024;102:105074. https://doi.org/10.1016/j.ebiom.2024.105074 38507877 PMC10960098

[8] Chour A , Basse C, Lebossé F, et al Management of sotorasib-related adverse events and hepatotoxicities following anti-PD-(L)1 therapy: Experience with sotorasib in two French anti-cancer centers and practical guidance proposal. Lung Cancer. 2024;191:107789. https://doi.org/10.1016/j.lungcan.2024.107789 38614068

[9] Garth S , Lichter A, Ratain M. Sotorasib, the Poster Child for Project Optimus: Truths and Fantasies. The ASCO Post. 2024.

[10] Katayama R , Khan TM, Benes C, et al Therapeutic strategies to overcome crizotinib resistance in non-small cell lung cancers harboring the fusion oncogene EML4-ALK. Proc Natl Acad Sci USA. 2011;108:7535-7540. https://doi.org/10.1073/pnas.1019559108 21502504 PMC3088626

[11] Kantarjian HM , Talpaz M, O’Brien S, et al Dose escalation of imatinib mesylate can overcome resistance to standard-dose therapy in patients with chronic myelogenous leukemia. Blood. 2003;101:473-475. https://doi.org/10.1182/blood-2002-05-1451 12393385

[12] Rea D , Etienne G, Corm S, et al Imatinib dose escalation for chronic phase–chronic myelogenous leukaemia patients in primary suboptimal response to imatinib 400 mg daily standard therapy. Leukemia. 2009;23:1193-1196. https://doi.org/10.1038/leu.2009.32 19242496

[13] Marusyk A , Janiszewska M, Polyak K. Intratumor heterogeneity: The rosetta stone of therapy resistance. Cancer Cell. 2020;37:471-484. https://doi.org/10.1016/j.ccell.2020.03.007 32289271 PMC7181408

[14] Malapelle U , Pisapia P, Pepe F, et al The evolving role of liquid biopsy in lung cancer. Lung Cancer. 2022;172:53-64. https://doi.org/10.1016/j.lungcan.2022.08.004 35998482

[15] Gadotti LL , Nogueira Amorim Canedo FS, Ribeiro MFSA, et al Successful drug rechallenge following severe acute alectinib-induced interstitial lung disease in a patient with advanced ALK-rearranged lung adenocarcinoma. Clin Lung Cancer. 2021;22:e481-e486. https://doi.org/10.1016/j.cllc.2020.06.021 32771343

[16] Ogasawara S , Chiba T, Ooka Y, et al Is intra-patient sorafenib dose re-escalation safe and tolerable in patients with advanced hepatocellular carcinoma? Int J Clin Oncol. 2014;19:1029-1036. https://doi.org/10.1007/s10147-014-0668-4 24519322

[17] Huang JR , Chou CW, Chao HS. Successful rechallenge of alectinib after remission of severe alectinib-induced interstitial lung disease. J Oncol Pharm Pract: Off Publ Int Soc Oncol Pharm Practit. 2021;27:1311-1314. https://doi.org/10.1177/1078155220961557 33054691

[18] Ngu S , Tse K, Chu MMY, Ngan HYS, Chan KKL. Olaparib dose re‐escalation in ovarian cancer patients who experienced severe and/or uncommon adverse events: a case series. Asia Pac J Clin Oncol. 2021;17:3-11. https://doi.org/10.1111/ajco.13584 33860646

[19] Koga T , Suda K, Fujino T, et al KRAS Secondary mutations that confer acquired resistance to KRAS G12C inhibitors, sotorasib and adagrasib, and overcoming strategies: Insights from in vitro experiments. J Thor Oncol: Off Publ Int Assoc Study Lung Cancer. 2021;16:1321-1332. https://doi.org/10.1016/j.jtho.2021.04.015 33971321

[20] Punekar SR , Velcheti V, Neel BG, Wong KK. The current state of the art and future trends in RAS-targeted cancer therapies. Nat Rev Clin Oncol. 2022;19:637-655. https://doi.org/10.1038/s41571-022-00671-9 36028717 PMC9412785

